# Small-Scale Habitat-Specific Variation and Adaptive Divergence of Photosynthetic Pigments in Different Alkali Soils in Reed Identified by Common Garden and Genetic Tests

**DOI:** 10.3389/fpls.2016.02016

**Published:** 2017-01-05

**Authors:** Tian Qiu, LiLi Jiang, ShanZhi Li, YunFei Yang

**Affiliations:** ^1^Key Laboratory of Vegetation Ecology, Ministry of Education, Institute of Grassland Science, Northeast Normal UniversityChangchun, China; ^2^School of Life Sciences, Changchun Normal UniversityChangchun, China; ^3^Key Laboratory of Molecular Epigenetics, Ministry of Education, Northeast Normal UniversityChangchun, China

**Keywords:** reed, alkali salt, leaf position, local adaptation, outlier loci, photosynthetic pigment, population genomics, selection

## Abstract

Flexibility of photosynthetic pigment traits is an important adaptive mechanism through which plants can increase mean fitness in a variable environment. Unlike morphological traits in plants, photosythesis has been shown to exhibit phenotypic plasticity, responding rapidly to environmental conditions. Meanwhile, local adaptation at small scales is considered to be rare. Thus, detecting the small-scale adaptive divergence of photosynthetic pigments presents a challenge. Leaf concentrations of photosynthetic pigments under stressful conditions may be reduced or maintained. Concentrations of some pigments and/or ratio of Chlorophyll a (Chla) to Chlorophyll b (Chlb) do not change markedly in some species, such as the common reed, *Phragmites australis*, a cosmopolitan grass and common invader. Little is known about photosynthetic responses of this plant to varying levels of alkali salt. Few studies have attempted to account for the relationship between pigment accumulation and leaf position in wild plant populations in grasslands. In this study, photosynthetic pigment concentrations and the total Chl(a+b)/Car ratio decreased as the growing season progressed and were shown to be significantly lower in the habitat with a higher soil pH value and less moisture when compared between habitats. The Chla/Chlb ratio did not differ significantly between habitats, although it increased significantly over time. Leaves in the middle position may be functionally important in the response to soil conditions because only pigment concentrations and the Chl(a+b)/Car ratio of those leaves varied between habitats significantly. The outlier loci, used to evaluate molecular signatures of selection, were detected by Arlequin, Bayescan, and Bayenv analyses. In the simulated habitats of common garden, the local genotypes had higher values of Chla, Chlb, Chl(a+b), Car in their home habitat than did genotypes originating from the other habitat. Q_ST_–F_ST_ comparisons provided evidence of divergent selection. It appears likely that soil moisture, pH and electric conductivity drove local adaptation. Combined approaches that utilize information on phenotypes from field and common garden experiments, genome-wide markers, and environmental data will be the most informative for understanding the adaptive nature of the intraspecific divergence.

## Introduction

Photosynthetic pigments are responsible for absorbing and transforming light energy (Biswal et al., 2012) and are involved in photoprotective processes and antioxidant activity, all of which contribute to effective biomass and oxygen production (Kuczynska et al., 2015). Chlorophyll a (Chla) is present in the reaction centers of photosystems I and II and in the pigment antenna, whereas chlorophyll b (Chlb) is found exclusively in the pigment antenna system. The Chla/Chlb ratio, which is related to antenna size, is an indicator of functional pigment equipment and light adaptation. The Chla/Chlb ratio is suggested to be a more important determinant of maximum fluorescence intensity than the chlorophyll (Chl) content (Dinç et al., 2012). A second class of pigments, the carotenoids (Car), are mainly involved in photoprotection and can act as antioxidants. A low value of the total Chl(a+b)/Car ratio can increase the efficiency of the photoprotective system under stress conditions (Ebrahimiyan et al., 2013).

The pigment concentration is strongly correlated with photosynthetic capacity and is an important indicator of growth status and photosynthetic properties (Cenzano et al., 2013). As a leaf functional trait, Chl content has been shown to be consistent with vegetative growth traits that are crucial individual fitness components (Bragato et al., 2009; Zhang et al., 2013). Chl content can also be indicative of stress injury and resistance. Several studies have documented that it is reduced under conditions of high irradiance, high altitude, high CO_2_ exposure, intense drought and pH levels >8 in both aquatic and terrestrial plants (Bragato et al., 2009; Hussner et al., 2015). However, the total Chl content and the Chl *a*/*b* ratio show no elevation-dependent trends in *Polylepis tarapacana*, which has been interpreted as an adaptive mechanism allowing the maintenance of its photosynthetic capacity (González et al., 2007; García-Plazaola et al., 2015).

The common reed [*Phragmites australis* (Cav.) Trin. ex Steud.] is one of the most widely distributed flowering plants on Earth and an invader in North America (Lambertini et al., 2008; Kirk et al., 2011). In *P. australis*, 20‰ salinity, high concentrations of heavy metals and low nitrogen supply, reduce aboveground growth and photosynthetic pigment concentrations (Lippert et al., 2001; Bragato et al., 2009; Eller et al., 2014). However, not all the pigments or traits change significantly in this species. Eleven geographically distinct clones from Europe and the United States, with the exception of a Swedish clone, displayed only minor differences in Chl(a+b) and total Car contents (Hansen et al., 2007). Among the concentrations of Chla, Chlb, total Chl(a+b) and total Car, only Chlb content was found to be significantly higher in introduced North American *P. australis* than in native its counterpart, although the shoot dry matter content of the invasive group was significantly higher (Guo et al., 2014). Similarly, among different habitats that were quantified and categorized according to soil properties, only the Chlb content of salt marsh was significantly lower than that from a mesophytic habitat, although the Chla and Chl(a+b) contents were similar (Nada et al., 2015).

**Figure 1:**
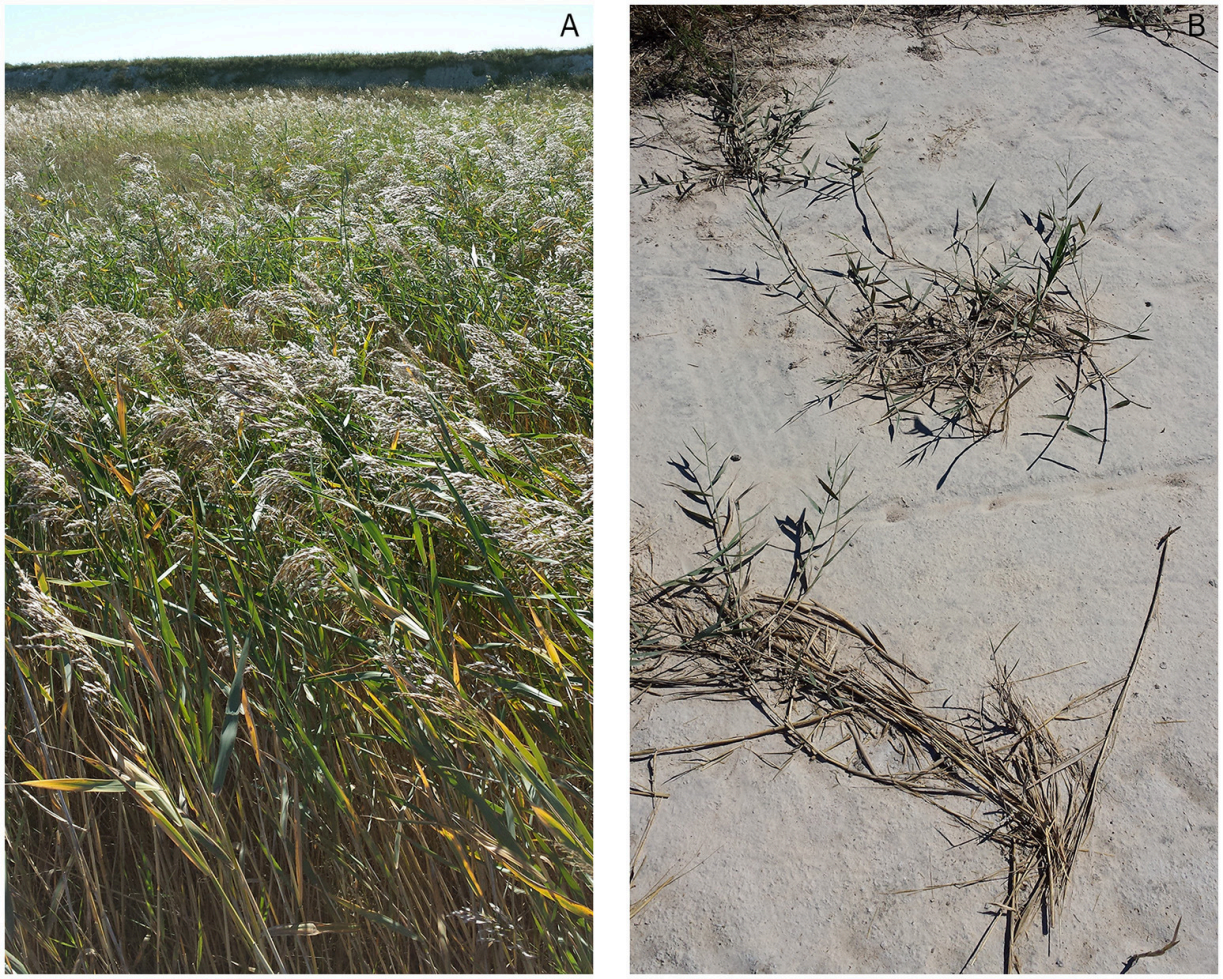
*****P. australis*** at natural sites at the Pasture Ecology Research Station of Northeast Normal University, Changling, Jilin Province, China. (A)** Habitat 1; **(B)** Habitat 2.

The Songnen Prairie in northeast China is one of the three largest soda saline-alkali areas in the world (Yu et al., 2014). Alkali stress causes injury to plants not only through salt stress involving osmotic and ionic stress but also through high pH. Previous reports have suggested that alkali salt can cause iron deficiency and the precipitation of Mg^2+^ as well as enhance the activity of the Chl-degrading enzyme chlorophyllase, which reduces photosynthetic pigment concentrations (Yang et al., 2011). However, little is known about the effects of alkali salt in *P. australis* (Chen et al., 2015), which grows and can even thrive in salinity/alkaline-eroded grassland in the Songnen Prairie. A mosaic of contiguous diverse habitats is present in this region due to differences in micro-terrain and levels of salt stress and/or alkali stress, and the local reed populations have evolved easily discernable phenotypes that are adapted to various habitats (Yang and Lang, 1998; Yang and Li, 2003; Liu and Yang, 2012). Thus, *P. australis* is not only a major forage grass but also a primary candidate for ecological restoration. In this study, reed individuals from two different dryland habitats with alkalized meadow soil, which differed mainly in soil pH, soil moisture and electric conductivity (EC), were investigated on a small scale; i.e., within an area of approximately 10 × 5 km. The dry mass, shoot length and density and rhizome length were much greater in *P. australis* from the seasonally waterlogged, low-lying meadow with a pH of 8–8.5 (Habitat 1, designated H1) than in the alkaline patch, which lacked accumulated rainwater and had a pH greater than 10 (Habitat 2, designated H2) (Figure 1). To understand whole-plant responses, further knowledge of the habitat-specific variation of photosynthetic pigments is required.

We hypothesized that these two habitats might strongly affect the pigment characteristics of *P. australis*. To test this hypothesis, the factors of leaf position and time course were considered. Leaf position has been reported to influence plant ontogenesis, various leaf traits, the contents of natural constituents, and tolerance to environmental stresses (Jullien et al., 2009; Ahmad et al., 2012; Vagiri et al., 2015). Few studies have attempted to account for the relationship between pigment accumulation and leaf position (Lippert et al., 2001; Juvany et al., 2013). Furthermore, a number of studies related to leaf position have been performed in both model and cultivated species (Ahmad et al., 2012; Song et al., 2012); however, little is known about wild populations in grasslands (Du and Yang, 1988; Peri et al., 2011). Samples of *P. australis* with leaves in various positions were collected at specific time intervals, and the mean values of measured characteristics from all of the leaves from each position represent plant-level values.

We previously observed significant genetic differentiation of *P. australis* between these two habitats (Qiu et al., 2016). The AFLP (amplified fragment length polymorphism) technique is one of the most suitable and efficient genome scanning approaches. It was applied using capillary gel electrophoresis, which is considered powerful and reliable with high resolution power, rapid analysis, and low sample consumption. This approach has been used to identify “outlier loci” and enables the evaluation of molecular signatures of selection (Nosil et al., 2009).

Because local adaptation is classically characterized by the higher fitness of local genotypes relative to genotypes from other habitats (“local vs. foreign” criterion; Kawecki and Ebert, 2004) and significant genotype × environment interactions, a common-garden experiment was conducted to disentangle the genetic and environmental effects on pigment traits and identify the selective agents driving local adaptation. The reproduction of standardized habitats with alkali salt under common-garden conditions offers an alternative to field transplants. If differences between field-collected populations persist under standard conditions, local adaptation is then implicated as the most important adaptive strategy. Interpretations of local adaptation can be challenged because forces other than selection (e.g., drift) can also lead to genetic differentiation (Kawecki and Ebert, 2004); thus, comparative evaluations of quantitative (Q_ST_) under common-garden conditions and neutral (F_ST_) estimators of genetic differentiation can be applied to test whether the observed population divergence exceeds that expected by genetic drift alone (Defaveri and Merilä, 2014).

Therefore, in this study, the following research topics were addressed. (1) Species-specific and habitat-specific variation of photosynthetic pigment traits of *P. australis* in alkalized meadow soil with varying alkaline levels were evaluated. The factors of leaf position and time course were considered. (2) We combine the analysis of phenotypes in common-garden conditions with genetic and environmental analyses to understand the adaptive divergence of photosynthetic pigments in *P. australis* on a small scale. As physiological processes, such as photosynthesis in *P. australis*, can, unlike other morphological traits, show considerable phenotypic plasticity in response to environmental conditions (Hansen et al., 2007), the analysis of common garden data can contribute to the evidence for or against local adaptation.

## Materials and methods

### Study area

The *P. australis* samples were collected within an approximately 10 × 5-km area of grassland at the Pasture Ecology Research Station of Northeast Normal University, Changling, Jilin Province, in the Songnen Prairie of China (123°45′E, 44°38′N). The region is characteristic of a semi-arid and semi-wet continental monsoon climate. The mean annual precipitation is 313–581 mm, 70% of which falls between June and September. Annual evapotranspiration is 3- to 4-fold the amount of precipitation. Mean daily temperatures greater than 10°C can persist for 120–140 days per year and can reach 21.5–23.6°C (Li et al., 2014). *Phragmites australis* often grows as a companion species in the alkalized meadow but forms monodominant communities in local low-lying areas or saline-alkaline patches (Yang and Li, 2003).

### Field samples and measurements

The two sampled groups of *P. australis* occur widely because the two corresponding habitats lie in a mosaic of various contiguous habitats (Figure 1). Six sampling sites from each habitat were selected (Figure S1, Table S1). The two dryland habitats can be described as follows: (1) In a seasonal waterlogged low-lying meadow (H1), the monodominant reed community occurs with coverage greater than 85%. The soil is alkalized meadow soil with a pH of approximately 8–8.5. Water typically accumulates seasonally during the rainy season. The reed grows well in a reasonably moist soil, and its companion species are mainly *Polygonum sibiricum, Scirpus planiculmis*, and *Echinochloa caudate*. (2) In an alkaline patch without seasonally accumulated rainwater (H2), a monodominant reed community has also appeared. The soil is alkalized with a pH greater than 10 and the nutrients in the surface layer of soil have been lost. The soil is hardened and is poorly permeable. The reeds grow sparsely and in tufts with short stems. The community coverage is less than 20%. However, after the rainy season, annual salt-tolerant plants or halophytes flourish, such as *Chloris virgata, Kochia scoparia, Suaeda heteroptera, Suaeda corniculata, Suaeda glauca*, leading to a community landscape dominated by these species at the end of the growing season (Yang and Li, 2003).

On July 15, July 25, August 5, and August 15, 2012, which corresponded to 60, 70, 80, and 90 days of plant growth, respectively, samples were collected. After August 15, the reeds began to produce inflorescences and senesce. Because relatively more leaves are present at the corresponding positions during the mid-growth phase, this phase is suitable for determining the influence of leaf position. We counted positions from the shoot base to the apex irrespective of leaf death. Chlorophyll a (Chla), chlorophyll b (Chlb), total chlorophyll (Chl(a+b)), and total carotenoid (Car) concentrations were determined spectrophotometrically from 100-mg aliquots of acetone extracts of freshly collected leaf laminae by using 80% acetone according to Lichtenthaler (1987). The pigment concentrations were expressed in mg/g of leaf fresh weight, with three replicates per site. Fully expanded fresh leaves from six other individuals at each site were collected randomly, dried using silica gel, and stored at −20°C for genetic analysis. All of the sampled individuals were separated by a minimum of 30 m to decrease the likelihood of sampling clone-mates. As other authors have shown, in the case of species in which clonal propagation is the main form of reproduction, maximizing the number of sampled sites rather than the number of shoots at each site is more informative of genetic diversity (Kirk et al., 2011). Minimizing the impact of reed collection on local soil erosion was also taken into consideration. Three soil samples (15-cm length of side, 15-cm depth) from the individual site corresponding to the common garden in the field were measured for soil moisture, pH and EC, which were the main soil properties according to which habitats were classified (Nada et al., 2015; Table 1). Soil moisture was determined by oven drying a known weight of each soil sample at 105°C until a constant weight was reached. Soil pH and EC were determined using a PHS-3C pH meter and a DDS-307 EC meter (Shanghai precision and scientific instrument, Shanghai, China) in a 1:5 soil-water solution (Li et al., 2014).

**Table 1:** **Soil properties of the two habitat conditions and the common garden conditions (mean ± ***SE***)**.

	**Soil moisture (%)**	**pH**	**Conductivity (us/cm)**
Habitat 1 (H1)	17.43 ± 1.57	8.20 ± 0.12	151 ± 9
Habitat 2 (H2)	5.76 ± 0.51	10.78 ± 0.07	649 ± 74
H1-H2 *P*	<0.001	<0.001	<0.001
Alkali stress treatment 1 (AS1)	42.42 ± 1.19	8.27 ± 0.09	352 ± 13
Alkali stress treatment 2 (AS2)	49.70 ± 0.70	9.76 ± 0.03	2355 ± 247
Alkali stress treatment 3 (AS3)	48.26 ± 0.87	9.94 ± 0.04	2425 ± 111
AS1-AS2 *P*	<0.001	<0.001	0.001
AS1-AS3 *P*	0.001	<0.001	<0.001
AS2-AS3 *P*	0.541	0.022	0.992

*The significance of the t-test and post-hoc comparisons are also shown*.

### AFLP genotyping

Genomic DNA was extracted from 72 individuals (36 from each habitat) using a modified cetyltrimethylammonium bromide (CTAB) method (Kidwell and Osborn, 1992). A standard AFLP analysis (Vos et al., 1995) was performed with minor modifications (Wang et al., 2005). Selective amplification primers fluorescently labeled at the 5′-end (Applied Biosystems Inc., Foster City, CA, USA) were used, along with an ABI-automated 3730XL DNA capillary sequencer. Restriction-ligation was conducted using an *Eco*RI/*Mse*I endonuclease mixture (New England Biolabs, Massachusetts, USA) and double-stranded adaptors. The four *Eco*RI+3/*Mse*I+3 primer pairs that provided the most reliable, consistently scorable bands were chosen after being screened for selective amplification (Table S2). A ROX-500-labeled internal size standard (Applied Biosystems) was added to each sample, and the software GeneMapper v.4.1 (Applied Biosystems) was utilized to collect and score the raw fluorescence data. We scored all well-resolved and reliable bands with a binary code, where zero represented an absent band, and one represented a band that was present. We excluded singleton observations from the dataset (i.e., markers with only one non-consensus band). One plant that continued to produce noisy sequencer electropherograms after the analyses were run several times was excluded. All scoring was performed “blindly” by the same person, who lacked any information about the samples. Non-overlapping peaks between 50 and 500 bp were included.

### Common garden experiment

The rhizomes of *P. australis* were collected in May 2012 from three sites (genotypes) distributed in the western, central and eastern areas of the research station for each habitat; these three sites were among the six sites established for the field samples (Figures S1, S2). Three clones per site for each habitat were taken to the campus of Northeast Normal University, Changchun, Jilin Province (125°18′E, 43°51′N). This region experiences a continental monsoon, and the climate is similar to that of the region where the research station is located. All of the rhizomes were physically separated from one other and cultivated in an experimental plot in the sand for 6 weeks to produce at least 50 asexual individuals from the nodes of rhizomes per site. Then, healthy rhizomes were cut into 10-cm lengths, each containing one node, and only those with a uniform height aboveground were randomly transplanted into plastic pots (38-cm diameter, 30-cm height, three rhizome blocks per pot) containing peat soil. Each clone includes three pots.

In June 2015, when the plants had been grown for 3 years, showing comparable vegetative development, possible environmental maternal effect was removed fully. The ramets were 8 weeks old, and 54 plants (3 genotypes × 2 habitats × 3 replications × 3 treatments) were randomly assigned to three levels of alkali salt treatment. Two alkali (NaHCO_3_ and Na_2_CO_3_) salts were selected based on the salt components of the salt-alkaline soils over much of northeast China and were mixed in a 9:1 molar ratio (NaHCO_3_:Na_2_CO_3_) (Li et al., 2010). The alkaline stress groups were labeled AS1–AS3. The stress treatments were applied every 6 days, with the salt concentration gradually increasing for 7 weeks from 30 to 700 and 750 mmol L^−1^ for treatment groups AS2 and AS3, respectively, thereby causing the soil pH to approach 10 and simulating H2. The AS1 group was continually treated with 30 mmol L^−1^ salt until the pH value approached 8.0–8.5 to simulate H1. All of the pots were sheltered from the rain and watered when necessary. The inter-pot distances were sufficient to prevent competition among the plants for light. The photosynthetic pigment characteristics were measured from the fourth-youngest leaves of the three tallest shoots per pot. The properties of soil samples from 3 pots per habitat and per treatment were measured (Table 1).

### Data analyses

Loci under selection present in the tails of the F_ST_ distribution generated across loci as a function of heterozygosity between habitats were identified based on F-statistics generated using Arlequin 3.5 (Excoffier and Lischer, 2010). A null distribution of F_ST_ close to the empirical distribution, which was originally developed by Beaumont and Nichols (1996), was acquired with 20,000 coalescent simulations and 100 simulated demes per group. A more rigorous identification of candidate loci was implemented in BayeScan 2.1, which directly calculates *q*-values for each locus (Foll and Gaggiotti, 2008). The sample size was set to 5000 and the thinning interval to 10 following 20 pilot runs. Then, the loci were split into two subsets, “selected” and “neutral.” Bayenv2 was run for 10^6^ iterations by four independent runs and testing for association with a single environmental variable using both a linear model and Spearman rank correlation while accounting for among-population structure through neutral parameterization by control data (Günther and Coop, 2013).

The photosynthetic data were analyzed using SPSS Statistics 17.0 (IBM, New York). Student's *t*-test was used to test for differences between habitats at the plant level and the leaf level, and soil property differences. The data were tested for normal distributions and variance homogeneity with the Levene test, and data were log-transformed where necessary. Two one-way ANOVA tests were conducted with sampling date and treatment as the fixed factor and the pigment traits and soil properties as dependent variables, respectively. When the overall variation in an ANOVA was significant, *post-hoc* comparisons among means were performed using Tukey's HSD test at α = 0.05.

Correlations between pigment concentrations in each of AS1 and AS2 or AS3 were tested. The one-way ANOVA tests were conducted with habitat as an independent variable in each treatment. The patterns of variation in pigment traits between habitats and among treatments and genotypes were analyzed by a General Linear Model (GLM) implemented using the UNIANOVA procedure of PASW Statistics 18 (formerly SPSS) (IBM, New York), with treatment and habitat as fixed factors and genotype, nested within habitats, as a random factor. The change in variability between habitats was described by calculating SSpop/SStotal, where SSpop is the sum of squares between the two habitats and SStotal is the total variability identified from the one-way ANOVA.

To test whether the among-habitat divergence in pigment characters was greater than expected if they evolved neutrally, the Q_ST_-F_ST_ approach was implemented (Defaveri and Merilä, 2014). We first decomposed the variation of each pigment trait, between habitats ($\sigma_{\text{B}}^{2}$) and within habitats ($\sigma_{\text{W}}^{2}$) in each treatment, by using the VARCOMP procedure (REML method) of PASW to calculate the observed Q_ST_ following eqn (1) ($\sigma_{\text{B}}^{2}$ in place of $\sigma_{\text{BE}}^{2}$). The $\sigma_{\text{W}}^{2}$ is the variance component among clones within sites. Then, the expected among-habitat variance component ($\sigma_{\text{BE}}^{2}$) and within-habitat variance component ($\sigma_{\text{W}}^{2}$) values were used to calculate the expected Q_ST_ of a neutral trait as follows:

(1) $$\begin{array}{l} {\text{Q}}_{\text{ST}}=\sigma_{\text{BE}}^{2}/\left(\sigma_{\text{BE}}^{2}+2\sigma_{\text{W}}^{2}\right) \end{array}$$

The $\sigma_{\text{BE}}^{2}$ of a neutrally evolving trait was obtained using F_ST_, which was calculated from the above-mentioned neutral loci and the$\sigma_{\text{W}}^{2}$ according to Equation (2).

(2) $$\begin{array}{l} \sigma_{\text{BE}}^{2}=\frac{2{\text{F}}_{\text{ST}}\sigma_{\text{W}}^{2}}{1-{\text{F}}_{\text{ST}}} \end{array}$$

We simulated the distribution of the test statistic, Q_ST_–F_ST_, 10,000 times, which is the null hypothesis of evolution in a neutrally evolving trait. We then tested if the observed Q_ST_–F_ST_ differed by the neutral expectations. The analyses were carried out in R using code from Lind et al. (2011).

## Results

### Habitat-specific variation of photosynthetic pigment traits

The Chla, Chlb, Chl(a+b), and Car contents and the Chl(a+b)/Car ratio of the plants in H2, alkaline patch without seasonally accumulated rainwater were 25.4, 25.6, 25.5, 13.7, and 12.6% lower, respectively, than those of the plants in H1, the seasonally waterlogged low-lying meadow (*P* < 0.001) (Figure S3). However, no significant differences in the Chla/Chlb ratio were observed (*P* = 0.444). Those photosynthetic pigment traits decreased markedly with increasing time in each habitat, whereas only the Chla/Chlb ratio showed the opposite trend (Figure S4). The H2 plants consistently presented lower values than did the H1 plants. The values of Chla, Chlb, Chl(a+b), Car and the Chl(a+b)/Car ratio in H1 declined by 36.3, 45.9, 38.5, 21.5, and 22.7%, respectively, compared with the values at the first sampling date. Similarly, the values of those traits in H2 decreased by 45.0, 52.1, 46.6, 27.0, and 27.6%, respectively. Thus, the larger changes over time in H2 relative to H1 were indicative of habitat differences. The decrease in Car content and the Chl(a+b)/Car ratio over time varied across these two habitats, as shown in Figure S4.

Comparisons of pigment traits at the same leaf position between the habitats revealed significant differences between pairs of middle leaves but no significant differences in the basal or apical leaf pairs (Table S3, Figure S3). Most of the pairs showing significant differences shifted gradually to relatively higher positions with time. The pigment traits generally exhibited the same changes as shown in Figures S3, S4 regardless of leaf position except for Car, for which stable values were obtained from the middle leaves of position 9 in H1 and position 7 in H2 (data not shown).

### Outlier detection

**Figure 2:**
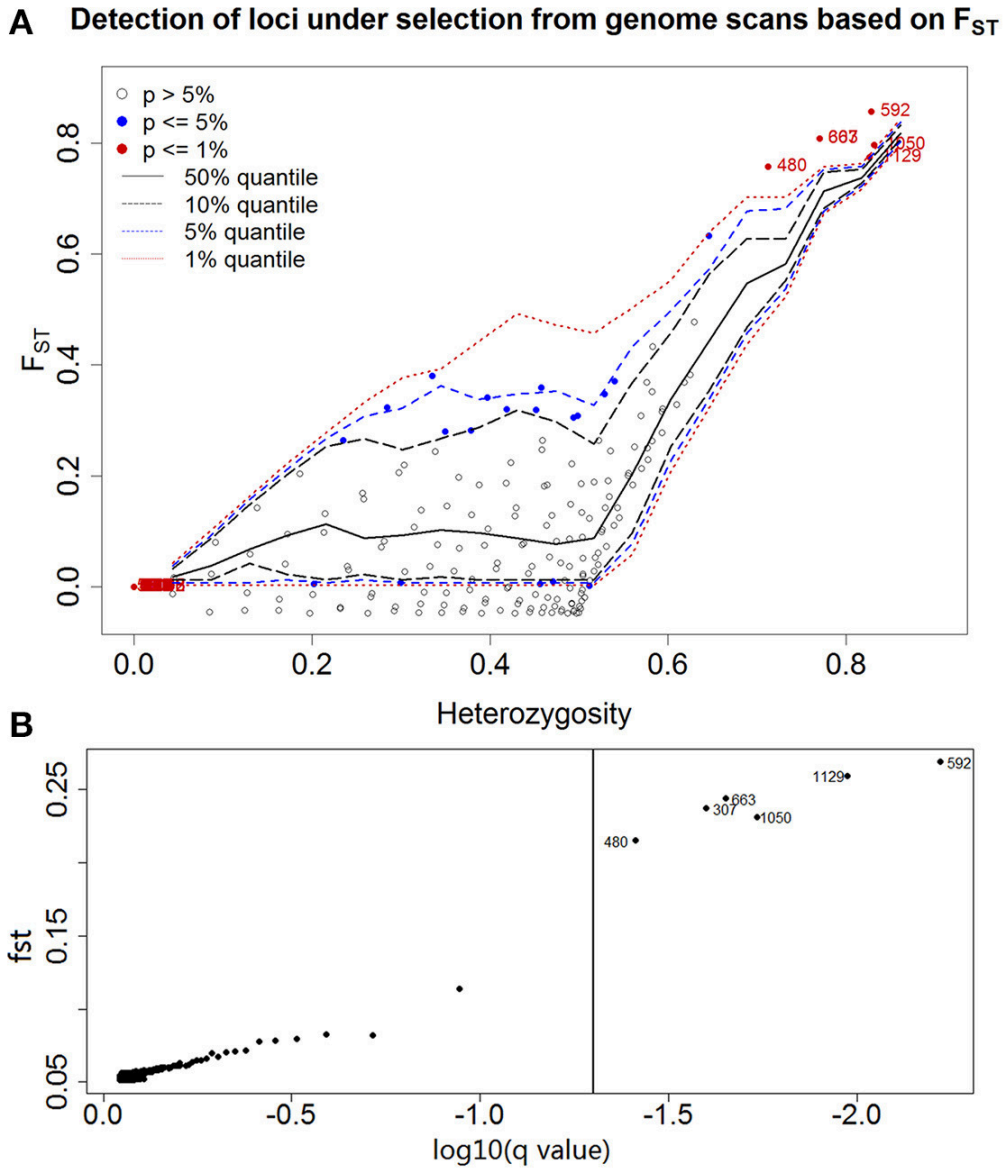
**Genomic scan of ***P. australis*** individuals conducted to identify outlier loci between habitats**. Each point corresponds to an AFLP marker (*N* = 1132). **(A)** A plot of the joint distribution of F_ST_ values and heterozygosity generated with Arlequin 3.5 is shown. One-sided confidence interval limits obtained from simulated data are shown as dashed lines. Loci significant at the 5 and 1% levels are shown as filled circles, whereas the numbers of the loci under selection at the 1% level are indicated as 307, 480, 592, 663, 1050, and 1129, although 307 and 663 overlapped. **(B)** A more rigorous identification conducted with BayeScan 2.1. F_ST_ estimates are plotted against the log-transformed *q*-value, which is the FDR analog of the *P*-value in the context of multiple testing. With a *q*-value lower than 5%, the outlier loci are beyond the vertical line, thus providing robust evidence in favor of selection that is considered decisive.

A total of 1132 AFLP fragments from 71 sampled individuals were unambiguously scored from primer combinations, with a relatively low scoring error rate of 0.99%. All of the six outlier loci identified (0.53% of the total) exhibited F_*ST*_ values that were markedly greater than expected (Figure 2, mean ± *SE* = 0.8000 ± 0.0140) and are therefore likely to be subject to directional selection or linked to loci under selection. The remaining 1126 loci exhibited lower levels of differentiation (mean ± *SE* = 0.0364 ± 0.0029). The outliers identified by Arlequin 3.5 and BayeScan2.1 were consistent. Bayenv was used to serve as a cross-check for consistency with those of different model approaches. The six outliers showed high Bayes factors in the top 1% of the BF-values (BF >3) and the high absolute values of ρ (|ρ|>0.4). Therefore, the signal can be considered to be robust. The six putative outliers also showed high absolute values of ρ (|ρ|>0.4) when the environmental variables without normalization were used (Table S4).

### Common garden experiment

**Figure 3:**
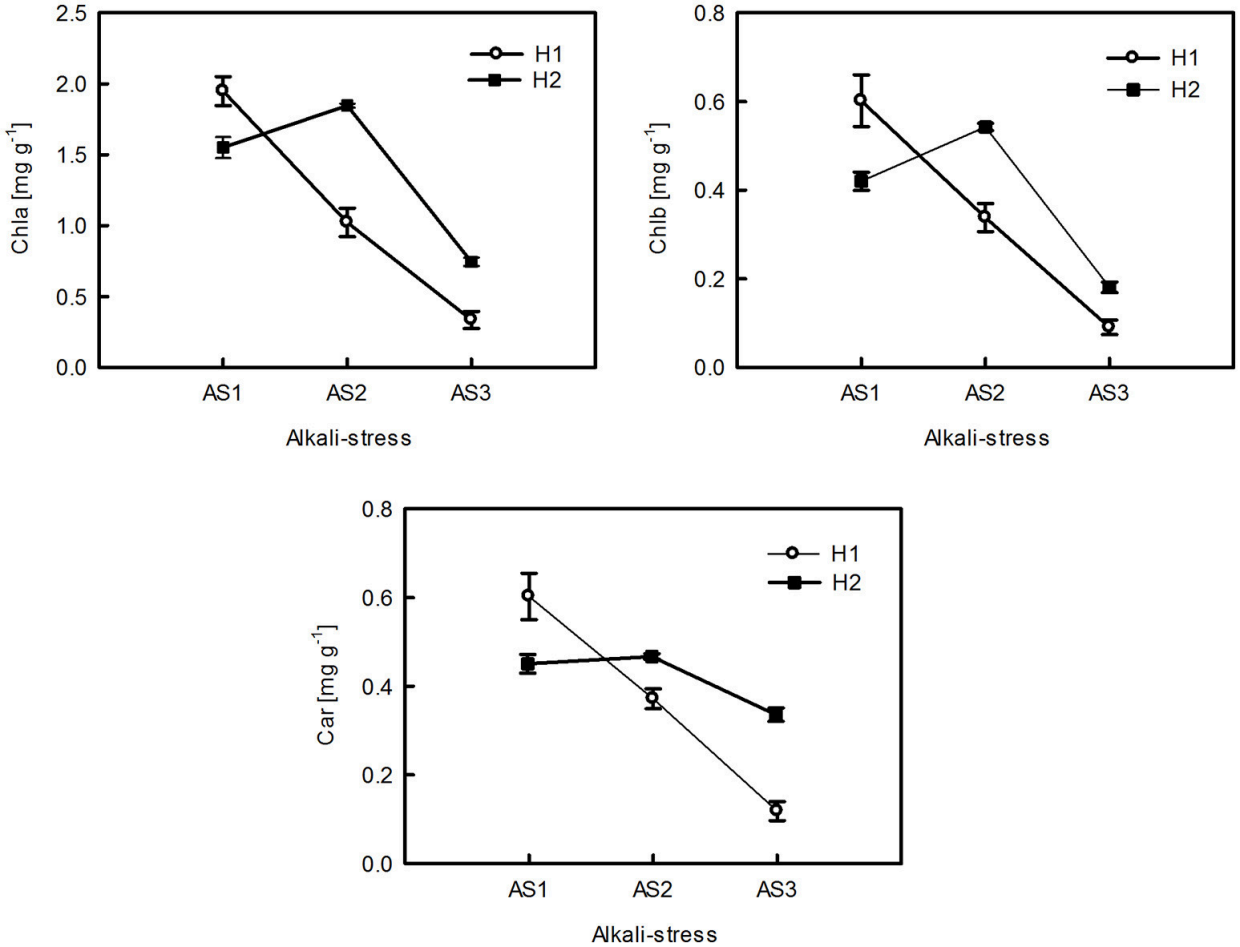
**Reaction norms of pigment concentrations of ***P. australis*** from the two habitats (H1, H2) under varying levels of alkali stress**. The data are the mean ± *SE* of nine replicates of three genotypes in each habitat.

**Figure 4:**
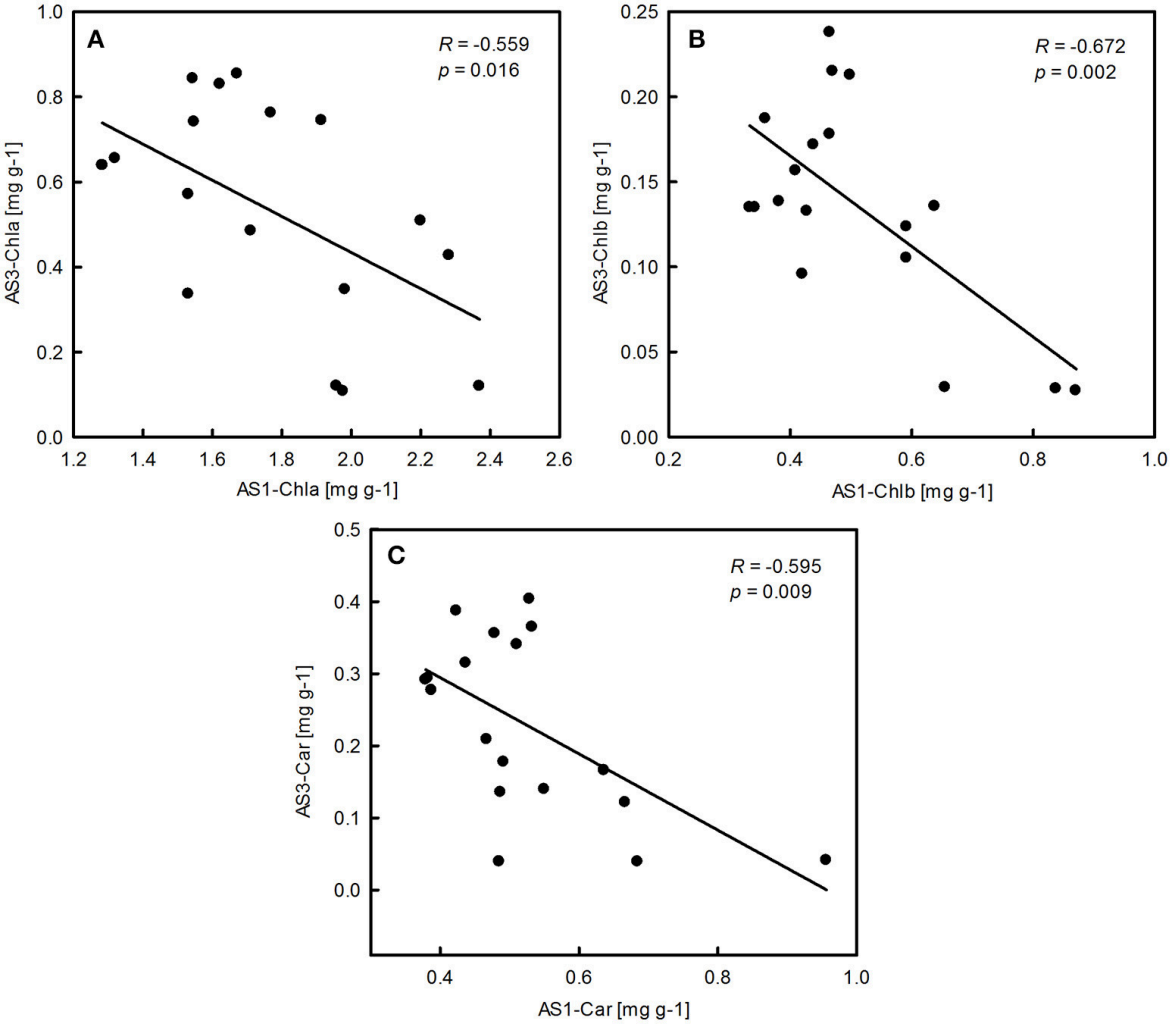
**Significant correlations between pigment concentration of ***P. australis*** in alkali stress treatment 1 (AS1) and alkali stress treatment 3 (AS3)**. Means are shown for each replication.

**Figure 5:**
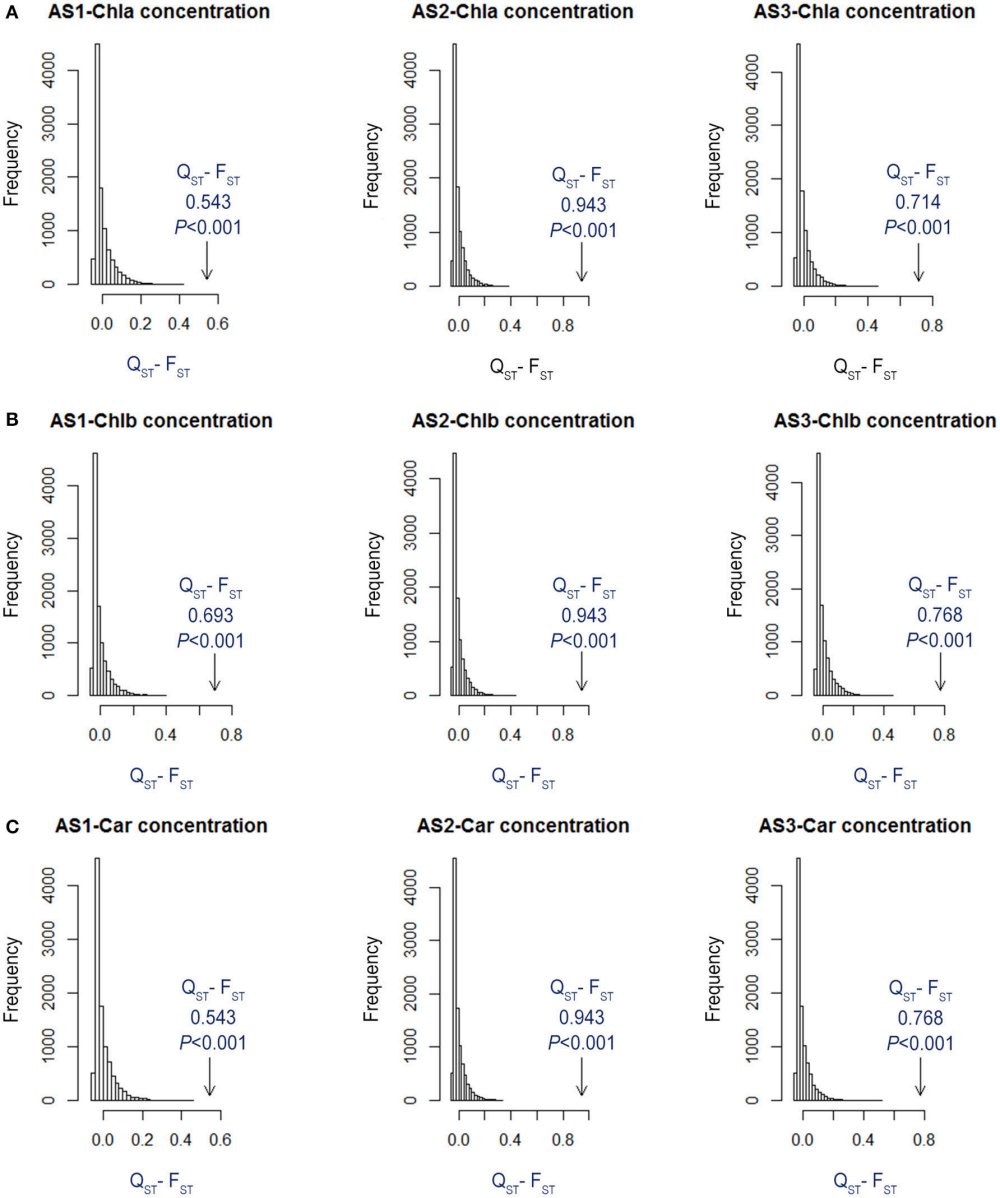
**Simulated distributions of Q_**ST**_-F_**ST**_ for neutrally evolving traits for (A)** Chla, **(B)** Chlb, and **(C)** Car from the two habitats within each alkali treatment (AS1, AS2, and AS3). The observed values of Q_ST_-F_ST_ are indicated with arrows, and *P*-values were calculated from the quantile of the simulated Q_ST_-F_ST_ distribution that had more extreme values than the observed Q_ST_-F_ST_ value.

The ANOVA tests revealed significant effects of habitat, alkali salt treatments, habitat-by-treatment and treatment-by-genotype interaction on most of the traits (Table 2) except Chl(a+b)/Car, for which no significant effect of habitat was found. No significant variation in any trait was found among genotypes. One-way ANOVA also revealed significant between-habitat variation in each treatment for all traits except Chl(a+b)/Car in the group AS1 (*P* = 0.872). Comparison of reaction norms showed that the local genotypes had higher values of Chla, Chlb, Chl(a+b), and Car in their simulated local habitat than did genotypes from the other habitat (Figure 3). These between-habitat pigment concentration differences were sustained not only during the common garden growth of group AS1 but also in group AS2 and AS3 under increasing pH, with larger percentages of variability between habitats in the latter two groups (Table 3). Soil moisture, pH and EC differed significantly between the two habitats (all *P* ≤ 0.001) (Table 1). The fitness trade-off (negative correlation) expected under a pattern of local adaptation was found in the groups AS1 and AS3 for Chla, Chlb and Car but not Chl(a+b)/Car (Figure 4). Chla, Chlb and Car within each treatment showed evidence of adaptive divergence between habitats, with Q_ST_–F_ST_ values significantly larger than the Q_ST_–F_ST_ values expected for a neutrally evolving trait (Figure 5). The data for Chl(a+b) are not shown as the patterns of changes in Chla and Chlb were the same.

**Table 2:** *****F***-values from ANOVA tests of the effects of treatment, habitat, genotype and the two-factor interactions on the pigment concentration in ***P. australis*****.

	**Habitat**	**Treatment**	**Genotype (habitat)**	Habitat ^*^ treatment	Treatment ^*^ genotype
Chla	10.170^*^	50.517^**^	1.55 ns	9.573^**^	2.761^*^
Chlb	11.074^*^	37.805^**^	0.488 ns	5.636^*^	3.509^**^
Car	16.432^*^	24.566^**^	0.585 ns	11.711^**^	3.018^*^
Chl(a+b)/Car	0.383 ns	47.685^**^	3.409 ns	35.774^**^	1.336 ns

*The ANOVA model incorporates treatment, habitat and genotype effects, with treatment and habitat as fixed effects and genotype nested within habitats as a random effect. Significance levels are indicated as follows: ns, non-significant*;

* 0.01 < P < 0.05; and

** *P < 0.01*.

**Table 3:** **Percentage of variability in pigment concentration between plants from the two habitats in each treatment**.

	**Alkali stress treatment 1 (AS1) (%)**	**Alkali stress treatment 2 (AS2) (%)**	**Alkali stress treatment 3 (AS3) (%)**
Chla	38.26	80.36	70.11
Chlb	35.07	70.94	56.06
Car	31.23	51.25	80.99

*Values are based on SS_pop_/SS_total_, where SS_pop_ is the sum of squares between the two habitats and SS_total_ is the total variability identified from one-way ANOVA tests for AS1, AS2, and AS3*.

## Discussion

### Variation of photosynthetic pigment traits between habitats

The flexibility of photosynthetic pigment traits is an important adaptive mechanism through which plant increase mean fitness to meet the challenges inherent in a variable environment (Aubin-Horth and Renn, 2009). In this study, photosynthetic pigment traits in two habitats with different severities of soil alkaline conditions and moisture were investigated. The Chla, Chlb, Chl(a+b), and Car contents and the Chl(a+b)/Car ratio were shown to be generally lower in Habitat 2 and decreased over time. These results were expected because Chl loss is often a common effect of stress and occurs during senescence (Dinç et al., 2012). The age-related variation of the Chla/Chlb ratio and TChl content appears to be an adaptive response to stress conditions (Ebrahimiyan et al., 2013; Zhang et al., 2013), as recently inferred for juvenile mastic trees and *P. australis* (Bragato et al., 2009; Juvany et al., 2013). Water stress, as one of the main causes of alkali stress, commonly causes a decrease in leaf Chl concentration in grasses, which helps reduce light absorption. Because only Chla/Chlb ratio did not differ significantly between habitats, the relative high value of Chla/Chlb ratio in Habitat 2 is an index of drought adaptation due to a smaller light-harvesting complex, making photosystem II less susceptible during stress (Caudle et al., 2014). The consistent patterns of Chl(a+b), Car and the Chl(a+b)/Car ratio were likely due to the lower rate of loss of Car than of Chl(a+b), which reflects an adaptive strategy because carotenoid plays an important role in photoprotection and antioxidation (Ebrahimiyan et al., 2013; Zhang et al., 2013). The information about increased antioxidant capacity under different stresses has been reported largely (Naudts et al., 2014; Xu et al., 2014, 2015). Decrease in antenna pigments such as Chl b suggested that plants experienced a process of antenna size readjustment. All of these mechanisms may act in coordination to allow successful survival for *P. australis*.

At the low and high relative leaf positions, the differences between habitats were generally small, whereas the differences at the middle leaf position were much larger (Table S3). This pattern may be due to the different physiological status of these leaves (Lippert et al., 2001). The upper leaves can neither accumulate mineral elements nor inhibit ion toxicity efficiently and show pigment contents to be enhanced, whereas the lower, older leaves undergo senescence, with gradual declines in their ion-transportability and photosynthetic capacities. In contrast, middle leaves are mature and may be functionally important for adaptation to stressful environments, considering their susceptibility to various stresses including N supply, herbicide, light intensity, tropospheric ozone and so on (Zhang et al., 2010; Yoon et al., 2011; Wang et al., 2014).

### Adaptive divergence of photosynthetic pigments

Speciation is often initiated by the adaptive divergence of populations occupying ecologically distinct environments (Endler, 1977; Hendry et al., 2002). Local adaptation to diverse environments is an important evolutionary step before divergence. Although local adaptation at a small scale has been considered rare due to high gene flow, it is a common result of the driving force of divergent selection at a small scale, as noted by Kawecki and Ebert (2004). Examples of this phenomenon have recently accumulated in species with high gene flow (Defaveri and Merilä, 2014), which are relevant for specific ecological processes involving elevated pH values in the soil (Bone and Farres, 2001). Because plants are generally sessile, and if environmental gradients are strong, gene flow might be non-random, all of these could thus generate adaptive genetic differentiation in quantitative characters at small spatial scales (Shapiro et al., 2006; Ross et al., 2009). It is therefore not surprising that this study found evidence for local adaptation.

Levels of genetic differentiation can be highly variable across a genome (Nosil et al., 2009). In the case of model organisms, it is easy to track the “outlier locus” under selection based on whole-genome information, whereas for non-model organisms, such as *P. australis*, it is difficult to identify candidate genes due to restricted genomic resources (Wang et al., 2012). AFLPs have frequently been used to scan genomes in non-model organisms. They can provide a large number of random loci scattered throughout the genome and detect candidate genomic regions without identifying loci of interest *a priori* (Herrera and Bazaga, 2008; Feng et al., 2016). The most commonplace approaches applied for AFLP genome scans have been Dfdist, Bayescan analyses until now (Yang et al., 2016). The six outlier loci were identified consistently by these two methods and examined by Bayenv method which provide a useful statistic for ranking markers by their allele frequency correlation with an environmental variable. Although the sampled habitat patches were not adjacent, the various patches among which the common reeds were distributed were parapatric. Thus, it appears reasonable to find that the proportion of the genome exhibiting outlier behavior (0.53%) at a small scale was relatively low relative to the range (0.4–9.0%) reported for outlier loci in previous studies (Nosil et al., 2009).

Although the field experiment provided confirmatory evidence of the differences in Chla, Chlb, Chl(a+b), Car, and the Chl(a+b)/Car ratio, the results of the common garden experiment did not provide robust evidence concerning Chl(a+b)/Car ratio. In the common garden experiment, we used population replication to rule out other forces (e.g., drift or migration) as the cause of differentiation as different samples from similar habitats should show similar responses to given environmental conditions if the differentiation is driven by those conditions (Defaveri and Merilä, 2014). Because the climatic conditions were very similar at the small scale considered here, soil properties should be the key factors responsible for selection regimes. It seems likely that soil conditions, especially soil moisture, pH and EC, determined the habitat-specific variation and drove local adaptation in photosynthetic pigment contents.

In conclusion, photosynthetic pigment concentrations and the Chl(a+b)/Car ratio in the field were shown to significantly decrease in the habitat with a higher soil pH value and less moisture. Only the Chla/Chlb ratio showed no alkali salt dependent trend in *P. australis*. The common garden experiment indicated that local adaptation can be maintained over small spatial scales. Additional support for this inference was provided by the population-genomic approach that detected outlier loci, and Q_ST_–F_ST_ comparisons. Soil moisture, pH and EC differentiated among the two habitats, which may have acted as selective agents to produce such an adaptive divergence.

## Author contributions

YY designed research; LJ contributed new reagents or analytical tools; TQ and SL performed research and TQ analyzed data; TQ and YY wrote the paper.

### Conflict of interest statement

The authors declare that the research was conducted in the absence of any commercial or financial relationships that could be construed as a potential conflict of interest.
