# Supplementary material for: Small-Scale Habitat-Specific Variation and Adaptive Divergence of Photosynthetic Pigments in Different Alkali Soils in Reed Identified by Common Garden and Genetic Tests
Source: Front Plant Sci. 2017 Jan 5;7:2016. doi: 10.3389/fpls.2016.02016 (PMC5216671; doi:10.3389/fpls.2016.02016)
Supplement: Supplementary file 1 [file DataSheet1.docx]

**Supplementary material**

**Fig.S1** Sampling locations of *Pragmites australis* in 2 habitats. Different habitats are indicated by distinct symbols (square, habitat 1 (H1); circle, habitat 2 (H2)) and those with asterisk were used in common garden.

**Fig.S2** *Phragmites australis* materials in the common garden. Rhizomes were collected (A) and cultivated in the sand (B). We cut the rhizomes into consistent length and randomly transplanted 3 per pot with uniform height of individuals (C).

**Fig.S3.** Effects of different habitats on the photosynthetic pigment characteristics of *P. australis*. The data are presented as the mean±SE. The t-test significance is shown in the inserts. The alkaline patch (H2) exhibited less water and a higher soil pH value (greater than 10) than the seasonally waterlogged, low-lying meadow (H1, pH 8 to 8.5). H1 denotes Habitat 1, and H2 denotes Habitat 2.

**Fig.S4.** Time course-dependent variation of pigment accumulation in *P. australis*. The data are presented as the mean±SE. Different letters indicate significant difference at the 5% level. H1 denotes Habitat 1, and H2 denotes Habitat 2.

**TableS1** Site locations and related geographical information.

| Site | Location information | Longtitude (E) | Latitude (N) |
| --- | --- | --- | --- |
| 1 | West H2 | 123.49 | 44.555 |
| 2 | West H2 | 123.494 | 44.558 |
| 3 | West H1 | 123.496 | 44.564 |
| 4 | West H1 | 123.496 | 44.553 |
| 5 | Middle H2 | 123.519 | 44.599 |
| 6 | Middle H2 | 123.525 | 44.596 |
| 7 | Middle H1 | 123.521 | 44.589 |
| 8 | Middle H1 | 123.532 | 44.587 |
| 9 | East H2 | 123.558 | 44.588 |
| 10 | East H2 | 123.557 | 44.582 |
| 11 | East H1 | 123.558 | 44.575 |
| 12 | East H1 | 123.568 | 44.574 |

H1：habitat 1；H2：habitat 2

**Table S2.** The adaptors, pre-selective primers and selective amplification primer pairs used for the AFLP analyses in this study.

|  | Sequence | | | | |
| --- | --- | --- | --- | --- | --- |
| Adaptor |  | | | | |
| *EcoR*Ⅰ-adaptor 1 | 5'-CTCGTAGACTGCGTACC-3' | | | | |
| *EcoR*Ⅰ-adaptor 2 | 5'-AATTGGTACGCAGTCTAC-3' | | | | |
| *Mse*Ⅰ-adaptor 1 | 5'-GACGATGAGTCCTGAG-3' | | | | |
| *Mse*Ⅰ-adaptor 2 | 5'-TACTCAGGACTCAT-3' | | | | |
| Pre-selective primer | | | | | |
| *EcoR*Ⅰ+A | 5'-GACTGCGTACCAATTCA-3' | | | | |
| *Mse*Ⅰ+C | 5'-GATGAGTCCTGAGTAAC-3' | | | | |
| *EcoR*Ⅰ+3 primer(without or with 5'-FAM) | | | | | |
| a. *E*-AAC | 5'-GACTGCGTACCAATTCAAC-3 | | | | |
| b*. E-*AAG | 5'-GACTGCGTACCAATTCAAG-3' | | | | |
| c. *E*-ACA | 5'-GACTGCGTACCAATTCACA-3' | | | | |
| i. *E*-AGA | 5'-GACTGCGTACCAATTCAGA-3' | | | | |
| *Mse*Ⅰ+3 primer | | | | | |
| 3. *M*-CAG | 5'-GATGAGTCCTGAGTAACAG-3' | | | | |
| 4. *M*-CAT | 5'-GATGAGTCCTGAGTAACAT-3 | | | | |
| 7. *M*-CTG | 5'-GATGAGTCCTGAGTAACTG-3' | | | | |
| primer combinations | | | | | |
| *EcoR*Ⅰ+3 | a | b | c | i |  |
| *Mse*Ⅰ+3 | 7 | 4 | 3 | 4 |  |

**Table S3.** Comparisons of the pigment characteristics of leaves at the same position and time course between habitats in *P. australis*. Significant results of *P*-values based on t-tests are indicated in bold at α=0.05.

| Leaf position | | | | | | | | | | | | |
| --- | --- | --- | --- | --- | --- | --- | --- | --- | --- | --- | --- | --- |
|  |  | 3 | 4 | 5 | 6 | 7 | 8 | 9 | 10 | 11 | 12 | 13 |
|  | Sampling date |  |  |  |  |  |  |  |  |  |  |  |
| Chla | Jul 15 | 0.970 | 0.372 | **0.008**** | **0.002**** | **0.001**** | **0.006**** | **0.001**** | **0.005**** |  |  |  |
|  | Jul 25 |  | 0.717 | **0.003**** | **0.008**** | 0.326 | **0.023*** | 0.091 | **0.045*** | 0.103 | 0.114 |  |
|  | Aug 5 |  | 0.782 | 0.938 | 0.245 | 0.065 | **0.009**** | **0.008**** | **0.009**** | 0.155 | 0.819 | 0.953 |
|  | Aug 15 |  |  |  | 0.441 | 0.092 | **0.005**** | **0.010*** | **0.013*** | **0.005**** | 0.19 | **0.001**** |
| Chlb | Jul 15 | 0.966 | 0.41 | **0.006**** | **0.016*** | **0.001**** | **0.015*** | **0.001**** | **0.007**** |  |  |  |
|  | Jul 25 |  | 0.588 | **0.003**** | **0.008**** | 0.29 | **0.017*** | 0.052 | **0.041*** | 0.154 | 0.069 |  |
|  | Aug 5 |  | 0.891 | 0.879 | 0.144 | **0.049*** | **0.011*** | **0.004**** | **0.005**** | 0.126 | 0.374 | 0.664 |
|  | Aug 15 |  |  |  | 0.713 | 0.154 | **0.006**** | **0.018*** | 0.125 | **0.044*** | 0.324 | 0.076 |
| Chl(a+b) | Jul 15 | 0.969 | 0.38 | **0.008**** | **0.002**** | **0.000**** | **0.008**** | **0.001**** | **0.005**** |  |  |  |
|  | Jul 25 |  | 0.686 | **0.003**** | **0.007**** | 0.317 | **0.021*** | 0.08 | **0.041*** | 0.112 | 0.101 |  |
|  | Aug 5 |  | 0.857 | 0.981 | 0.215 | 0.06 | **0.009**** | **0.007**** | **0.007**** | 0.144 | 0.696 | 0.888 |
|  | Aug 15 |  |  |  | 0.488 | 0.1 | **0.005**** | **0.01*** | **0.009**** | **0.008**** | 0.211 | **0.003**** |
| Car | Jul 15 | 0.251 | 0.066 | **0.027*** | 0.064 | **0.001**** | **0.007**** | **0.004**** | **0.008**** |  |  |  |
|  | Jul 25 |  | 0.878 | 0.868 | 0.787 | 0.666 | 0.440 | 0.376 | 0.352 | 0.465 | 0.562 |  |
|  | Aug 5 |  | 0.149 | 0.444 | 0.232 | 0.112 | **0.018*** | 0.173 | 0.141 | 0.936 | 0.120 | 0.959 |
|  | Aug 15 |  |  |  | **0.049*** | 0.057 | **0.01*** | **0.016*** | **0.025*** | **0.000**** | **0.022*** | **0.000**** |
| Chl(a+b)/Car | Jul 15 | 0.27 | 0.395 | 0.915 | 0.281 | **0.05*** | 0.415 | **0.01*** | 0.131 |  |  |  |
|  | Jul 25 |  | 0.451 | 0.089 | 0.115 | 0.315 | **0.029*** | 0.056 | 0.117 | 0.285 | 0.297 |  |
|  | Aug 5 |  | 0.473 | 0.766 | 0.259 | 0.073 | 0.076 | **0.004**** | **0.036*** | 0.095 | 0.077 | 0.877 |
|  | Aug 15 |  |  |  | 0.334 | 0.491 | **0.021*** | **0.037*** | 0.101 | 0.693 | 0.783 | 0.833 |

**TableS4** A summary of putative F_ST_ outliers using Arlequin, Bayescan and Bayenv analyses.

| Locus | ARLEQUIN | BAYESCAN | BAYENV |  |  |
| --- | --- | --- | --- | --- | --- |
|  | (F_ST_ *P*-value) | (q-value) | (Bayes factor)^a^ | (\|ρ\|)^b^ | (\|ρ’\|)^c^ |
| 307 | 1.00E-07 | 0.0251 | * | ** | ** |
| 480 | 6.64E-04 | 0.0386 | * | ** | ** |
| 592 | 1.00E-07 | 0.0060 | * | ** | ** |
| 663 | 1.00E-07 | 0.0224 | * | ** | ** |
| 1050 | 1.00E-07 | 0.0184 | * | ** | ** |
| 1129 | 1.00E-07 | 0.0106 | * | ** | ** |

^a^ * Bayes factor > 3.

^b^ ** |ρ|＞0.4.

^c^ ** |ρ’|＞0.4, ρ’ was calculated by environmental variables without normalization.
