# Supplementary figures and images for: Small-Scale Habitat-Specific Variation and Adaptive Divergence of Photosynthetic Pigments in Different Alkali Soils in Reed Identified by Common Garden and Genetic Tests
Source: Front Plant Sci. 2017 Jan 5;7:2016. doi: 10.3389/fpls.2016.02016 (PMC5216671; doi:10.3389/fpls.2016.02016)

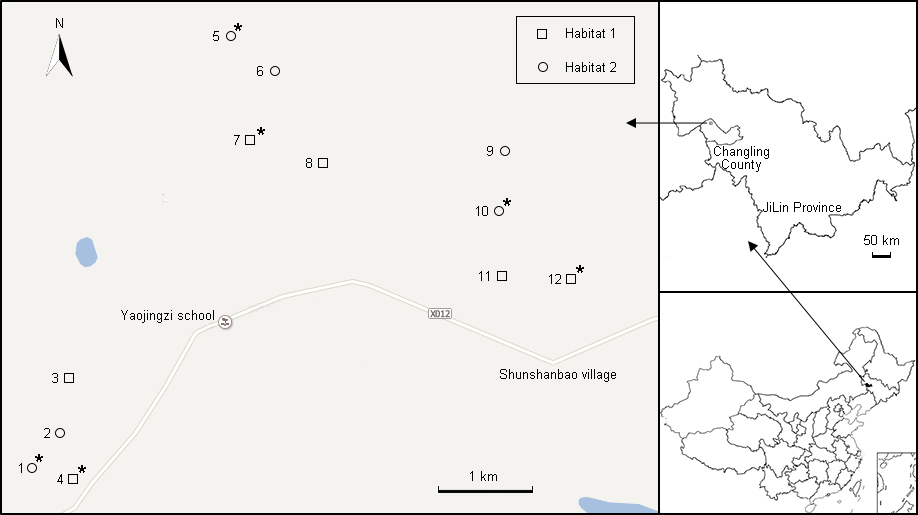

Supplement: Supplementary file 2 [file Image1.TIF]

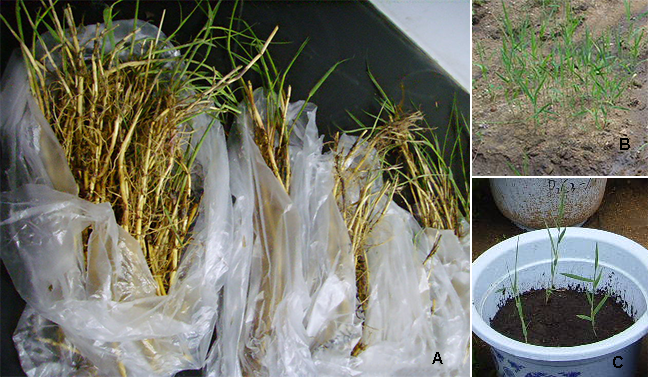

Supplement: Supplementary file 3 [file Image2.TIF]

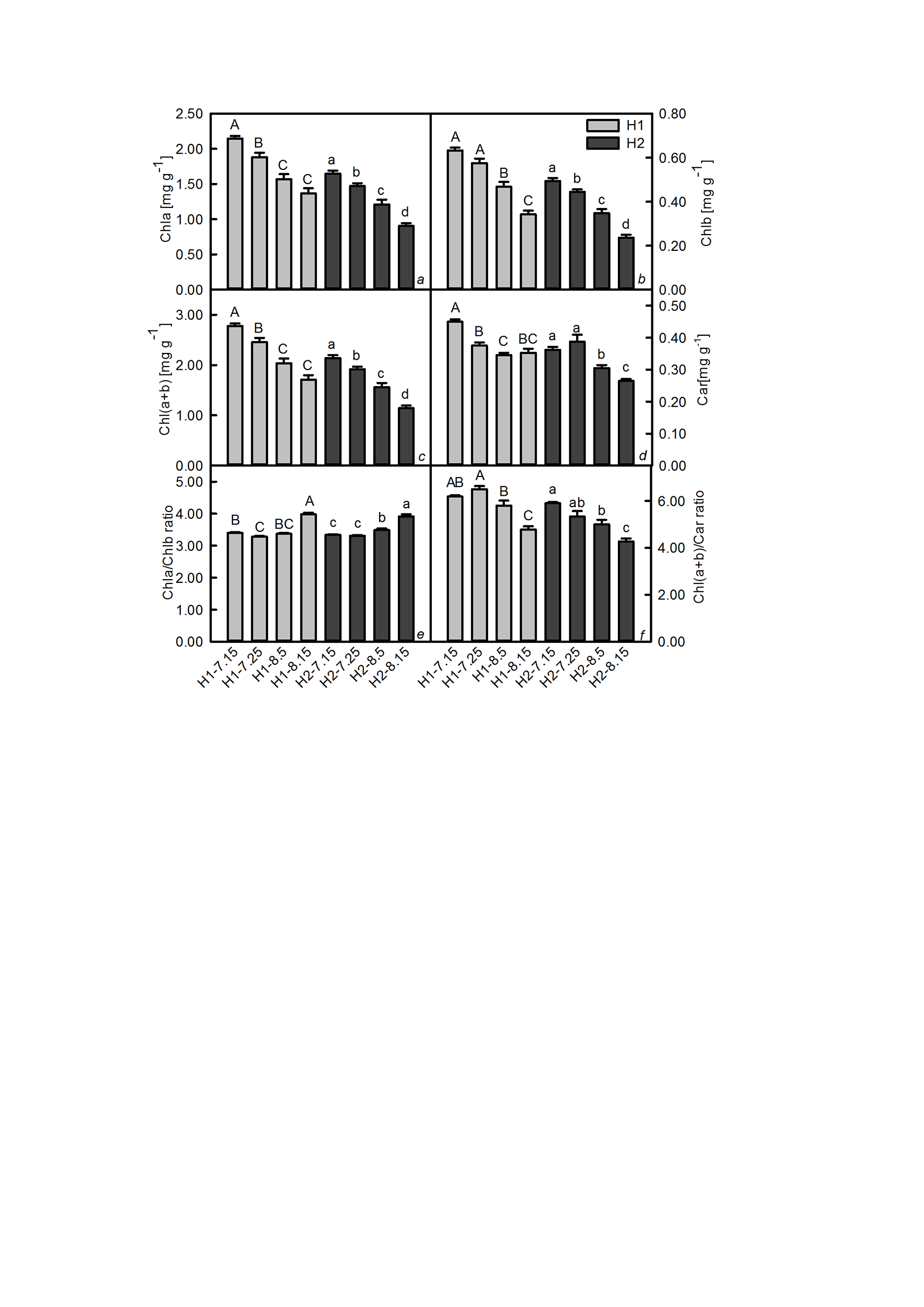

Supplement: Supplementary file 5 [file Image4.TIF]
